# Donor-Specific Anti-Human Leukocyte Antigen Antibodies Predict Prolonged Isolated Thrombocytopenia and Inferior Outcomes of Haploidentical Hematopoietic Stem Cell Transplantation

**DOI:** 10.1155/2017/1043836

**Published:** 2017-04-16

**Authors:** Xiaosu Zhao, Xiangyu Zhao, Mingrui Huo, Qiaozhen Fan, Xuying Pei, Yu Wang, Xiaohui Zhang, Lanping Xu, Xiaojun Huang, Kaiyan Liu, Yingjun Chang

**Affiliations:** ^1^Peking University People's Hospital and Peking University Institute of Hematology, Beijing Key Laboratory of Hematopoietic Stem Cell Transplantation, No. 11 South Street of Xizhimen, Xicheng District, Beijing 100044, China; ^2^Collaborative Innovation Center of Hematology, Peking University, Beijing, China; ^3^Peking-Tsinghua Center for Life Sciences, Beijing 100871, China

## Abstract

Prolonged isolated thrombocytopenia (PT) after allogeneic stem cell transplantation (allo-SCT) has a great impact on transplant outcome. In this study, we performed a retrospective analysis to investigate the association of donor-specific anti-human leukocyte antigen (HLA) antibodies (DSAs) with PT in 394 patients who underwent unmanipulated haploidentical blood and marrow transplantation (HBMT). For HLA antibody positive samples with a median fluorescent intensity (MFI) > 500, DSAs were further examined. A total of 390 patients (99.0%) achieved sustained myeloid engraftment. Of the 394 cases tested, 45 (11.4%) were DSA positive. The cumulative incidence of PT in this cohort of patients was 9.9 ± 1.5%. The incidence of PT was higher in patients with a MFI ≥ 1000 compared with those with a MFI < 1000 (16.8 ± 6.4% versus 7.4 ± 1.4%, *P* = 0.05). Multivariate analysis showed that the presence of DSAs (MFI ≥ 1000) was correlated to PT (hazard ratio (HR) 3.262; 95% confidence interval (CI), 1.339–7.946; *P* = 0.009) and transplant-related mortality (HR 2.320; 95% CI, 1.169–4.426; *P* = 0.044). Our results, for the first time, suggest an association of DSAs with PT after unmanipulated HBMT. It would help screen out the suitable donor and guide intervention. This indicated that DSAs should be incorporated in the algorithm for unmanipulated HBMT.

## 1. Introduction

For patients with hematologic malignancies, allogeneic stem cell transplantation (allo-SCT) is a kind of curative treatment [1–3]. Recently, haploidentical SCT provides alternative treatment options for patients lacking human-leukocyte antigen- (HLA-) matched related or unrelated donors. However, prolonged isolated thrombocytopenia (PT), which is defined as the engraftment of all peripheral blood cell lines other than a platelet (PLT) count ≥ 20 × 10^9^/L or dependence on PLT transfusions for more than 90 days after allo-SCT, has a great impact on transplant outcomes, especially in haploidentical SCT settings. The incidence of PT is around 5 to 37% after transplantation [4–6]. In our center, we established an unmanipulated haploidentical blood and marrow transplantation (HBMT) protocol that has a lower incidence of graft failure compared to other haploidentical transplant modalities [7], but PT still significantly increases the risk of transplant-related mortality (TRM) [4–6, 8]. Although the impaired PLT production and accelerated peripheral destruction are known to be the major causes of PT [4–6], there still might be other undiscovered factors that remain to be clarified [9].

Donor-specific antibodies (DSAs) are the anti-human leukocyte antigen (HLA) antibodies that specifically respond to the mismatched antigen of donor [10–12]. Several researchers, including us, have confirmed the effects of DSAs on graft failure (GF), including graft rejection (GR) and poor graft function (PGF), in patients who underwent haploidentical SCT either with T cell depletion or with T cell replete [13–15]. However, there is no data on the relationship of DSAs with PT after haploidentical SCT. Here, we performed a retrospective analysis to investigate the association of DSAs with the occurrence of PT in patients who underwent unmanipulated HBMT.

## 2. Materials and Methods

### 2.1. Patients

The consecutive patients who received unmanipulated HBMT from March 2010 to March 2014 at Peking University Institute of Hematology were enrolled in this study. All cases underwent DSA examination and had the complete data of DSA before transplantation. The transplant protocol was approved by the Institutional Review Board of Peking University People's Hospital, and the IRB approval number is 2012-27. The clinical trial registration number is NCT01617473. All patients signed informed consent forms. This study was conducted in accordance with the Declaration of Helsinki. The characteristics of patients and donors were shown in Table 1.

### 2.2. Transplant Protocol

The unmanipulated HBMT was performed as previously described [16, 17]. Patients were conditioned with busulfan (BU, 0.8 mg/kg iv, q6h), cyclophosphamide (CTX, 1.8 g/m^2^/d for 2 days), and antithymocyte globulin (rabbit ATG, Sang Stat, Lyon, France) (2.5 mg/kg/d iv for 4 days) or total body irradiation (TBI, 7.7 Gy), CTX, and ATG. All patients received G-CSF-mobilized bone marrow (BM) and peripheral blood stem cell transfusion. Cyclosporine A, mycophenolate mofetil, and short-term methotrexate were used for prophylaxis of graft-versus-host disease (GVHD).

### 2.3. Anti-HLA Antibody and DSA Examination

The patients and donors underwent HLA allele typing of at least the A, B, and DRB1 loci routinely. The examination was performed as previously [15]. In brief, patient plasma/serum was screened for class I and class II HLA antibodies with a LABScreen Mixed Kit (One Lambda, Canoga Park, CA, USA). The samples were incubated with mixed HLA class I- and class II-coated microspheres for 30 min in the dark and then washed before being incubated with anti-human immunoglobulin G-conjugated fluorescein isothiocyanate as described above for the first incubation. Finally, the samples were examined by a Luminex 200 flow analyzer (Luminex, Austin, TX, USA), and the data were analyzed with the HLA Fusion 3.2 software (One Lambda). The MFI of anti-HLA antibodies was obtained using the formula: sample beads − negative control beads. The samples with a MFI > 500 were further tested for the specificity of the antibody (DSA), using a LABScreen Single Antigen Kit (One Lambda).

### 2.4. Definitions of Engraftment

Neutrophil engraftment was defined as achieving an ANC more than 0.5 × 10^9^/L for three consecutive days, and platelet recovery was defined as achieving a platelet count more than 20 × 10^9^/L without platelet transfusions for seven consecutive days. Chimerism analysis was performed by DNA fingerprinting for short-tandem repeats in blood samples and/or chromosome fluorescence in situ hybridization of BM samples [18]. Full donor chimerism was defined as ≥95% leukocytes of donor origin in peripheral blood samples. Mixed chimerism was defined as more than 5% but less than 95% leukocytes of donor origin.

Primary GF consists of GR and PGF. GR is the failure to engraft neutrophils by day +28 for three consecutive days and the absence of donor hematopoiesis. PGF was defined as the presence of three cytopenic counts (ANC ≤0.5 × 10^9^/L, platelet ≤20 × 10^9^/L, and hemoglobin (HGB) ≤80 g/L) beyond day +28 with a complete donor chimerism in the absence of severe GVHD or hematological relapse. PT was defined as the engraftment of all peripheral blood cell lines other than a PLT count ≥20 × 10^9^/L or dependence on PLT transfusions for more than 90 days after allo-HSCT in the presence of complete donor chimerism. Patients with evidence of PGF or hematologic relapse within 90 days after transplantation were excluded.

### 2.5. Statistical Analysis

The reference date of June 30th, 2016, was used to define the end of follow-up. The median follow-up was 796 (range: 25–2309) days. The Fisher exact test or Wilcoxon test was used for two-group comparisons. Disease-free survival (DFS), TRM, and overall survival (OS) were calculated according to Kaplan-Meier statistics. Death before +90 d, GR, and PGF were considered a competing risk for PT. The difference of PT between groups was tested according to Gray's method, using R software for statistical computing. A two-sided *P* value of 0.05 was considered significant. The log-rank test was used for comparisons of Kaplan-Meier curves. Potential prognostic factors for PT, OS, DFS, relapse, and TRM were examined using Cox proportional hazards models. SPSS 22.0 software was used (Mathsoft, Seattle, WA, USA) for statistical analysis.

## 3. Results

### 3.1. The General Clinical Characteristics of Patients

Total of 394 patients were enrolled in this study. The median age of patients was 26 years (range, 2–58 years). All patients received a myeloablative conditioning regimen. The median dose of infused CD34^+^ cells was 2.61 (0.39–16.82) × 10^6^/kg. Except for patient age (*P* = 0.020), cases with PT and those without PT had equivalent patient and donor characteristics (Table 1).

### 3.2. Engraftment

Three hundred ninety patients achieved myeloid engraftment except for 4 patients (1.0%). The median time to neutrophil engraftment was 13 days (range, 8–27 days). Up to the follow-up, the incidence of platelet engraftment was 91.9% and the median time to platelet engraftment was 17 days (range, 6–265 days). 32 patients did not meet the criteria of platelet engraftment. Total of 4 patients developed GR and 39 patients (9.9%) were PGF. Among the patients who met the criteria of PGF, 9 patients died before +90 days and 16 patients later met the criteria of PT. In patients who met the criteria of PT, 16 patients only met the criteria of PT but not PGF. Thus, the cumulative incidence of PT in this cohort of patients was 9.9 ± 1.5%.

### 3.3. HLA Antibodies and DSA

Among all the patients, there were 99 (25.1%) with positive anti-HLA antibody, consisted of 48 males and 51 females. Of these positive patients, 63 (16.0%) had antibodies against HLA class I antigens and 57 (14.5%) had antibodies against HLA class II antigens. 31 (7.9%) patients had HLA antibodies against both classes I and II. In all HLA antibody positive patients, 45 (45.5%) had positive DSA. Of the 394 cases tested, 45 (11.4%) were DSA positive. Because we previously showed that high antibody titers of DSAs (MFI ≥ 10,000) were correlated to primary GR (*P* < 0.001) and that low antibody titers of DSAs (MFI ≥ 2000) were strongly associated with primary PGF (*P* = 0.005) [15], here, we analyzed the impacts of DSAs (MFI ≥ 1000) and DSAs (MFI ≥ 2000) on PT. Of the DSA positive patients, the MFI of 37 (9.4%) patients were more than 1000 and the MFI of 31 (7.9%) patients were more than 2000. The detailed information about HLA antibodies and DSA was shown in Table 2.

### 3.4. Relationship of DSAs with PT after Unmanipulated HBMT

The percentages of cases with positive anti-HLA antibody were 9.2% (27/295) in patients with good engraftment, 18.8% (6/32) in patients with PT, 30.8% (12/39) in patients with PGF, and 100% in patients with GR (Table 2). By using a competing risk analysis, the incidence of PT was higher in patient with a MFI ≥ 1000 compared with those with a MFI < 1000 (16.8 ± 6.4% versus 7.4 ± 1.4%, *P* = 0.05). For the competing events including death before +90 d, GR, and PGF, the incidence of PT was higher in patients with a MFI ≥ 1000 compared with those with a MFI < 1000 (29.9 ± 7.7% versus 6.5 ± 1.3%, *P* < 0.001). Univariate analysis showed that factors including elder (*P* = 0.020), infused lower dose of CD34^+^ cells (*P* = 0.061), positive HLA antibody (*P* = 0.092), and HLA class II antibody (*P* = 0.056) and DSAs (MFI ≥ 1000, *P* = 0.058) were associated with PT after unmanipulated HBMT (Table 3). However, multivariate analysis showed that the presence of DSAs (MFI ≥ 1000) (hazard ratio (HR) 3.262; 95% confidence interval (CI), 1.339–7.946; *P* = 0.009) was independently associated with PT. It also seemed that an infused lower dose of CD34^+^ cells might predict a higher incidence of PT (HR 0.481; 95% CI, 0.232–1.001; *P* = 0.05, Table 4).

### 3.5. Relationship of DSAs with Transplant Outcomes

To further investigate the relationship of DSAs with other transplant outcomes including TRM, relapse, DFS, and OS, we also took the above factors into the univariate and multivariate analyses. In the univariate analysis, DSAs (MFI ≥ 1000, *P* = 0.076), grade II–IV acute GVHD (*P* = 0.033), infused CD4^+^ (*P* = 0.024) and CD3^+^ cells (*P* = 0.088), donor-recipient relationship (*P* = 0.097), and disease status at the transplantation (*P* = 0.029) were associated with TRM. The results of multivariate analysis showed that an infused higher dose of CD4^+^ cells and DSAs (MFI ≥ 1000) were the independent risk factors of TRM (Table 4). For relapse, disease status (*P* < 0.001), sex match (*P* = 0.09), and HLA-DP (*P* = 0.024) were brought into multivariate analysis. Finally, patients in high risk and HLA-DP were related to leukemia relapse (Table 4). For DFS, chronic GVHD (*P* = 0.011), disease status (*P* < 0.001), and sex match (*P* = 0.094) were conducted into multivariate analysis. As our expected, patients in high risk and with chronic GVHD were associated with DFS (Table 4). It showed that with chronic GVHD (*P* = 0.006), disease status (*P* < 0.001) and DSAs (MFI ≥ 1000, *P* = 0.09) were related to OS in univariate and multivariate analyses though DSAs (MFI ≥ 1000) did not show a statistical significance (Table 4).

### 3.6. Effects of PT on Transplant Outcomes

**Figure 1:**
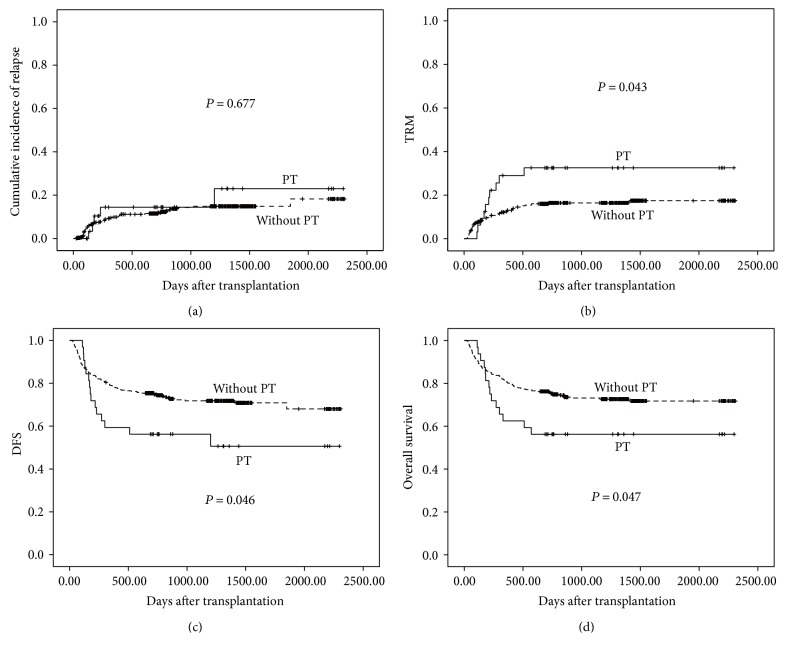
PT and transplant outcomes after haploidentical transplantation. (a) Cumulative incidence of relapse (CIR) and (b) TRM. (c) Disease-free survival. (d) Overall survival.

Compared to cases with PT, patients without PT had lower incidence of TRM (16.3 ± 0.02% versus 32.5 ± 0.09%, *P* = 0.043), similar incidence of relapse (12.3 ± 0.02% versus 14.4 ± 0.07%, *P* = 0.677), and higher probabilities of DFS (74.7 ± 0.02% versus 56.3 ± 0.88%, *P* = 0.046) and OS (75.2 ± 0.02% versus 56.3 ± 0.88%, *P* = 0.047) (Figure 1). Multivariate analysis showed that the onset of PT was independently associated with TRM (HR, 2.717; 95% CI, 1.343–5.498; *P* = 0.005). Besides, patients with PT seemed also to have a worse DFS (HR, 1.695; 95% CI, 0.984–2.921; *P* = 0.057) and OS (HR, 1.629; 95% CI, 0.928–2.858; *P* = 0.089).

## 4. Discussion

The association of DSAs with graft failure has been demonstrated by other researchers and us in either HLA-matched unrelated donor transplant, umbilical cord blood transplantation, or haploidentical transplantation [10–12, 15, 19]. In this study, we, for the first time, found an association of the presence of DSAs with PT after unmanipulated HBMT, indicating that DSAs may be involved in the pathogenesis of this complication. In addition, patients with the onset of PT-experienced inferior transplant outcomes provide further evidence suggesting that the presence of DSAs should be incorporated in the donor selection algorithm [20, 21].

The definition of a threshold for DSAs, according to MFI, is a premise for analyzing the association of DSAs with PT after transplantation. In previous studies, several cutoff values, such as MFI >500, 1000, 1500, 2000, and 5000, have been defined as DSA positive that was associated with graft failure in different transplant modalities with different conditioning regimens and GVHD prophylaxis [10, 11, 14, 15, 22]. In this study, the cutoff value of DSA MFI was 1000, which was associated with the onset of PT after unmanipulated HBMT. Our previous observation indicated that patients with DSA MFI ≥ 10000 experienced graft rejection and cases with DSA MFI ≥ 2000 experienced PGF. Our results suggest that high, intermediate, and low antibody titers of DSAs may lead to GR, PGF, and PT, respectively [15]. The fact that patients with PT experienced higher cumulative incidence of TRM and inferior DFS and OS supports the logical theory that the presence of DSAs results in PT, which may contribute to inferior survival.

Barge et al. [23] have found that DSAs may kill donor cells through antibody-dependent cell-mediated cytotoxicity (ADCC). In vitro experiment performed by us showed that DSAs could induce apoptosis of CD34^+^ cells and endothelial progenitor cells (EPCs) in the allografts (unpublished data). Kong et al. [24] from our center reported that the PT patients exhibited remarkable decreases in cellular elements of the vascular microenvironment of the bone marrow, including EPCs and perivascular cells, compared to the cases with good graft function after transplantation and the healthy donors, respectively. A multivariate analysis revealed that EPCs were an independent risk factor for PT. Their data suggested that an impaired BM vascular microenvironment may contribute to the occurrence of PT after allo-SCT [24]. It is reasonable that DSAs may lead to BM vascular microenvironment impairment via inducing apoptosis of EPCs, although further studies are needed. Our group also demonstrated that the recruitment of CD8^+^ T cells into BM might explain the suppression of megakaryocyte apoptosis through the elevated expression of CX3CR1^+^ in PT after allo-SCT [25]. These results indicate that an immune-mediated mechanism may contribute to the pathogenesis of PT after unmanipulated HBMT.

The present study had several limitations. First, our study is a single-center retrospective study. A prospective study with a training group and validation group is needed. Second, the present study only investigated the unmanipulated HBMT modality with an ATG conditioning regimen. Therefore, our results should be further confirmed in a multicenter clinical trial and in T cell-depleted haploidentical SCT modalities or in T cell-replete haploidentical SCT settings with postcyclophosphamide [26].

In conclusion, our results not only, for the first time, suggested an association of DSAs with PT after unmanipulated HBMT but also confirmed the effects of PT on inferior transplant outcomes. In addition, our study is clinically relevant and provides further evidence that DSAs should be incorporated in the algorithm for unmanipulated HBMT.

## Figures and Tables

**Table 1:** Patient and donor characteristics.

Characteristics	*n* = 394
Median age (range), years	26 (2–58)
Male sex, *n* (%)	231 (58.6%)
Diagnosis	
AML, *n* (%)	160 (40.6%)
ALL, *n* (%)	133 (33.8%)
CML, *n* (%)	22 (5.6%)
MDS, *n* (%)	41 (10.4%)
Others, *n* (%)	38 (9.6%)
Disease status, SR (%)	301 (76.4%)
Conditioning regimen	
MA, *n* (%)	394 (100%)
Number of HLA-A, B, DR mismatched, *n* (%)	
0	3 (0.8%)
1	22 (5.6%)
2	87 (22.1%)
3	282 (71.6%)
Donor-recipient sex match, *n* (%)	
Male-male	149 (37.8%)
Male-female	102 (25.9%)
Female-male	84 (21.3%)
Female-female	59 (15%)
Donor-recipient relationship, *n* (%)	
Father-child	150 (38.1%)
Mother-child	57 (14.5%)
Sibling-sibling	117 (29.%)
Child-parent	58 (14.7%)
Others	12 (3.0%)
ABO matched, *n* (%)	
Matched	224 (56.9%)
Major mismatched	74 (18.8%)
Minor mismatched	22 (5.6%)
Bidirect mismatched	74 (18.8%)
Cell compositions in allografts, median (range)	
Infused nuclear cells, 10^8^/kg	8.23 (1.78–23.69)
Infused CD34+ cells, 10^6^/kg	2.61 (0.39–16.82)
Infused lymphocytes, 10^8^/kg	2.93 (0.16–9.49)
Infused CD3+ cells, 10^8^/kg	2.0 (0.1–5.93)
Infused CD4+ cells, 10^8^/kg	1.1 (0.15–3.94)
Infused CD8+ cells, 10^8^/kg	0.69 (0.05–2.47)
Infused CD14+ cells, 10^8^/kg	1.48 (0.19–6.13)
aGVHD	
Grade 0-I aGVHD	259 (65.7%)
Grade II–IV aGVHD	135 (34.3%)
cGVHD	
No cGVHD	266 (67.5%)
cGVHD	128 (32.5%)

**Table 2:** The HLA-antibodies and DSA in different statuses of engraftment.

	Good engraftment	PT	PGF	GR
With positive anti-HLA antibody (*n*, %)	27/295 (9.2%)	6/32 (18.8%)	12/39 (30.8%)	4/4 (100%)
Median DSA MFI	3096	9374	4843	17214
Range of DSA MFI	504–12969	1403–18950	600–11736	3793–19948

**Table 3:** Patient characteristics in groups with and without PT.

	PT	Without PT	*P* value
*N*	32	362	
Patient age, median (range)	36 (3–54)	26 (2–58)	**0.020**
Patient sex, male, *n* (%)	17 (53.1)	214 (59.1)	0.320
Diagnosis, *n* (%)			0.510
AML	13 (40.6)	147 (40.6)	
ALL	14 (43.8)	119 (32.9)	
CML	1 (3.1)	21 (5.8)	
MDS	3 (9.4)	38 (10.5)	
Others	1 (3.1)	37 (10.2)	
Disease risk, *n* (%)			
SR	22 (68.8)	279 (77.1)	0.288
HR	10 (31.3)	83 (22.9)	
HLA incompatibility, *n* (%)			0.533
0 locus	0	3 (0.8)	
1 locus	2 (6.3)	20 (5.5)	
2 loci	4 (12.5)	83 (22.9)	
3 loci	26 (81.3)	256 (70.7)	
Donor-patient relation, *n* (%)			0.260
Sibling donor	9 (28.1)	108 (29.8)	
Father donor	4 (12.5)	53 (14.6)	
Mother donor	9 (28.1)	141 (39.0)	
Children donor	9 (28.1)	49 (13.5)	
Others	1 (3.1)	11 (3.0)	
Donor-patient sex match, number (%)			0.722
Male to male	11 (34.4)	138 (38.1)	
Male to female	11 (34.4)	91 (25.1)	
Female to male	6 (18.8)	78 (21.5)	
Female to female	4 (12.5)	55 (15.2)	
ABO matched, *n* (%)			0.213
Matched	15 (46.9)	209 (57.7)	
Major mismatched	8 (25.0)	66 (18.2)	
Minor mismatched	5 (15.6)	69 (19.1)	
Bidirect mismatched	4 (12.5)	18 (5.0)	
Cell compositions in allografts, *n* (%)			
Infused nuclear cells ≥ median	15 (46.9)	181 (50.0)	0.735
Infused CD34^+^ cells ≥ median	11 (34.4)	187 (51.7)	**0.061**
Infused lymphocytes ≥ median	13 (48.1)	160 (50.3)	0.829
Infused CD3^+^ cells ≥ median	14 (51.9)	160 (50.3)	0.878
Infused CD4^+^ cells ≥ median	16 (59.3)	159 (50.0)	0.356
Infused CD8^+^ cells ≥ median	16 (59.3)	157 (49.4)	0.324
Infused CD14^+^ cells ≥ median	16 (59.3)	157 (49.4)	0.324
Grade II–IV aGVHD	12 (44.4)	105 (33.0)	0.229
cGVHD	8 (25.0)	120 (33.2)	0.320
HLA antibody positive	12 (37.5)	87 (24.0)	**0.092**
HLA-I antibody positive	6 (18.8)	57 (17.9)	0.579
HLA-II antibody positive	8 (25.0)	49 (13.5)	**0.056**
HLA-DP antibody positive	3 (9.4)	29 (8.0)	0.732
DSA positive	6 (18.8)	39 (10.8)	0.174
MFI > 1000	6 (18.6)	31 (8.6)	**0.058**
MFI > 2000	4 (12.5)	27 (7.5)	0.310

AML: acute myeloid leukemia; ALL: acute lymphoblastic leukemia; CML: chronic myeloid leukemia; MDS: myelodysplastic syndrome; SR: standard risk; HR: high risk; aGVHD: acute graft-versus-host disease; cGVHD: chronic graft-versus-host disease; HLA-I/II: class I/II HLA antibody; HLA-DP: class I and II HLA antibody double positive; DSA: donor-specific antibody; DSA-DP: class I and II DSA double positive; MFI: median fluorescent intensity.

Bold means variates included in the multivariate analysis since their *P* value < 0.1 in the univariate analysis.

**Table 4:** Multivariate analysis of factors associated with transplant outcomes.

	HR	95% CI	*P* value
Prolonged isolated thrombocytopenia			
Infused CD34+ cells ≥ median	0.481	0.232–1.001	0.050
DSA			
MFI ≥ 1000	3.262	1.339–7.946	0.009
TRM			
Disease status	1.753	(0.985–3.118)	0.056
Infused CD4+ cells ≥ median	1.899	(1.079–3.340)	0.026
DSA			
MFI ≥ 1000	2.320	(1.169–4.426)	0.044
Relapse			
Disease status	3.882	(2.103–7.166)	<0.001
HLA-DP	2.552	(1.139–5.742)	0.0230
DFS			
Disease status	2.667	(1.761–4.038)	<0.001
cGVHD	0.622	(0.423–0.916)	<0.016
OS			
Disease status	2.504	(1.643–3.817)	<0.001
cGVHD	0.612	(0.413–0.908)	0.015
DSA			
MFI ≥ 1000	1.220	(0.998–2.138)	0.082
